# Who cares for syphilis? A cross-sectional study on diagnosis and treatment of syphilis by GPs in Amsterdam, the Netherlands

**DOI:** 10.3399/bjgpopen20X101027

**Published:** 2020-04-29

**Authors:** Michel Baas, Erna Beers, Alje P van Dam, Jan EAM van Bergen

**Affiliations:** 1 General Practice, Amsterdam UMC - Location AMC, Amsterdam, Netherlands; 2 Microbiologist, Public Health Service (GGD), Amsterdam, Netherlands; 3 Microbiologist, Amsterdam UMC - Location AMC, Amsterdam, Netherlands; 4 SOA AIDS Nederland, Amsterdam, Netherlands; 5 National Institute for Public Health and the Environment (RIVM), Bilthoven, Netherlands

**Keywords:** primary health care, sexually transmitted diseases, syphilis, Netherlands, general practitioners

## Abstract

**Background:**

Syphilis is a re-emerging infection. Sexually transmitted infection (STI) clinics and GPs are important providers of STI care in the Netherlands. The role of GPs in syphilis care is assumed to be small, since most men who have sex with men (MSM) visit STI clinics for STI care.

**Aim:**

To explore the role of GPs in the diagnosis and treatment of syphilis.

**Design & setting:**

Data on syphilis diagnostics by GPs in Amsterdam between 2011 and 2017 were retrieved from laboratories, covering 90% of the GPs. The study also used the academic GPs’ network database to explore the management of syphilis by GPs between 2013 and 2018.

**Method:**

Syphilis tests requested by GPs were analysed and compared with annual reports of the STI clinic. Patients with an International Classification of Primary Care-1 (ICPC-1) syphilis code were identified in the GP database. Cases diagnosed by the GP were evaluated whether they were treated by the GP or referred to secondary care.

**Results:**

In the laboratory database, GPs had diagnosed syphilis 522 times, compared with 2515 times by the STI clinics. Based on the 90% coverage of GPs, the contribution of all Amsterdam’s GPs was 19% of the total number of diagnoses. Consequently, the annual incidence of syphilis diagnosed by the GP was 10.2 per 100 000 inhabitants. Of the 43 cases identified in the GP database, six (14.0%) were referred and 33 (76.7%) were treated by a GP.

**Conclusion:**

Although for an individual GP, syphilis is rare to diagnose, GPs in Amsterdam do contribute to the rate of syphilis diagnosis and appear to treat the majority of cases that they have diagnosed.

## How this fits in

The role of GPs in syphilis care was assumed to be minimum, however the data show that the role of GPs in diagnosis and treatment of syphilis cannot be neglected. As syphilis is re-emerging, proper attention in primary care is warranted. Patients with syphilis in general practice need comprehensive STI care, including pre-exposure prophylaxis (PrEP) counselling.

## Introduction

Syphilis has been re-emerging in the Netherlands for several years.^1^ In 2017, the incidence of new syphilis infections surpassed the number of new HIV infections. Also, in 2017, 1228 cases of syphilis were reported by the National Institute for Public Health and the Environment (RIVM). Ninety-five per cent of all infections were in MSM. Reasons mentioned for this increased incidence are the increased prevalence of chemsex (sex under the influence of drugs), condomless sex, and increased popularity of dating sites and apps, and public sex environments.^2–5^

Symptoms of syphilis vary for different stages of the infection. Syphilis can cause serious complications to all organ systems, for instance, to the cardiovascular or nervous system. Complications may occur directly as well as years after infection. Thereby, syphilis increases the susceptibility and infectivity for HIV.^6^ Early detection and treatment of syphilis can prevent progression of the disease, as well as preventing mother-to-child transmission and limit further transmission in the population.

STI care in the Netherlands is provided by GPs and STI clinics; GPs carry out two-thirds of all STI consultations.^1^ GPs diagnose 79% of all STIs, and one-third of all new HIV infections. However, the number of syphilis cases is entirely based on data from STI clinics, since the number of syphilis diagnoses in general practice is unknown.

The assumption is that most MSM visit STI clinics for STI testing and, as a result, the role of the GP is limited in syphilis care.^7,8^ However, not every patient with syphilis is an MSM, and MSM might visit their GP for STI testing as well. In addition, patients can consult their GP with various symptoms of syphilis, not being aware of a possible STI.

The aim of this study was to explore the contribution of the GP in syphilis diagnoses and treatment.

## Method

### Study design

Two databases were used to study the role of the GP in diagnosing and treating syphilis. First, to study the contribution of the GP in syphilis diagnosis, a laboratory database containing all STI diagnostics by GPs in Amsterdam was used. Second, to gain insight into who is treating the patients with syphilis detected in primary care, the academic GPs’ network database was used.

### Setting

#### Laboratory data

Anonymous data of seven laboratories in Amsterdam where GPs request their diagnostics were aggregated. All major laboratories shared their data, resulting in an estimated 90% coverage of all STI diagnostics by GPs in Amsterdam. Tests in the same patient could be linked using anonymous patient numbers. Extracted from the database were all syphilis tests between 1 January 2011 and 1 January 2018, together with test date, test result, patient number, age, and sex.

#### Academic GPs’ network database

Data of the General Practice Department of Amsterdam University Medical Center (location AMC [Academic Medical Centre]) research database was used. This database contains 129 183 active patients aligned to eight different primary care centres. Six practices are located in southeast Amsterdam and two in the city centre, both areas are considered as high prevalence areas for STIs.^9,10^ Many non-Western migrants reside in southeast Amsterdam, and many MSM live in Amsterdam’s city centre.

Every consultation is documented as a free-text journal and coded with an ICPC-1 code; diagnostic records are stored as well. Multiple consultations on the same topic can be grouped in the patient file around one ICPC-1 code. Data were collected from all patients with consultations coded as lues: ICPC-1 codes Y70/X70, between 1 July 2013 and 1 July 2018. To determine the stage of disease and gain insight into the diagnostics and treatment process, data were collected 3 months before diagnosis and during 3-months follow-up. Extracted were: date of diagnosis, age, sex, HIV status, episode journals, syphilis diagnostics, and antibiotic prescriptions.

#### Case definition

A diagnosis of syphilis was identified in the laboratory database if one or more conditions were present:

1. Positive polymerase chain reaction (PCR).
2. Positive screening test (anti-*T* *reponema* *p* *allidum* or *T. pallidum* particle agglutination assay [TPPA] or *T. pallidum* hemagglutination assay [TPHA] and titre (venereal disease research laboratory [VDRL] or rapid plasma reagin [RPR]) ≥1:4.
3. Fourfold increase in VDRL or RPR (reinfection).

A diagnosis of syphilis was identified in the academic GPs’ network database if one or more conditions were present:

1. Positive PCR.
2. Positive screening test (anti-*T. pallidum* or TPPA or TPHA) and titre (VDRL/RPR) ≥1:4.
3. Fourfold increase in VDRL/RPR (reinfection).
4. Episode journal describing a new syphilis infection.

### Measurement

#### Laboratory data

Excluded from analysis were duplicates, tests with no patient identification number, failed tests or tests without the test result. All remaining syphilis tests were analysed and classified according to the diagnostic criteria. Primary outcome was the number of diagnoses and the GP’s contribution in diagnosing syphilis in Amsterdam compared with annual reports of the STI clinic. Secondary outcomes were the number of laboratory tests, lab requests, and positivity rates.

One laboratory test was one of the following: PCR, anti-*T. pallidum*, TPPA, TPHA, VDRL, or RPR. One lab request was defined as all laboratory tests in the same patient within a time frame of 30 days. This distinction was made to increase accuracy of positivity rates. Reinfections were counted if a separate lab request (>30 days) had a positive PCR test or fourfold increase in titre. Screening tests that were not otherwise specified were coded as anti-*T. pallidum*.

#### Academic GPs’ network database

All potential cases were evaluated manually for inclusion according to the case criteria. People aged ≤15 years and cases diagnosed at the STI clinic or elsewhere were excluded. Cases diagnosed by the GP were evaluated if they were treated by the GP or referred to secondary care for treatment. If patients were treated by the GP, the medication that was prescribed was evaluated. Only cases containing information about the treatment process (referral to secondary care or X-amount of penicillin injections) were evaluated for treatment or otherwise reported as missing data.

### Analysis

Frequencies and analysis were performed using IBM SPSS Statistics (version 24). Laboratory data were reported annually. Academic GPs’ network database data were aggregated across the study period.

### Data access

In order to perform this study, investigators had full access to the anonymous dataset that was retrieved from the database. Only the relevant data during the study period were accessible.

## Results

### Laboratory data

**Figure 1.**
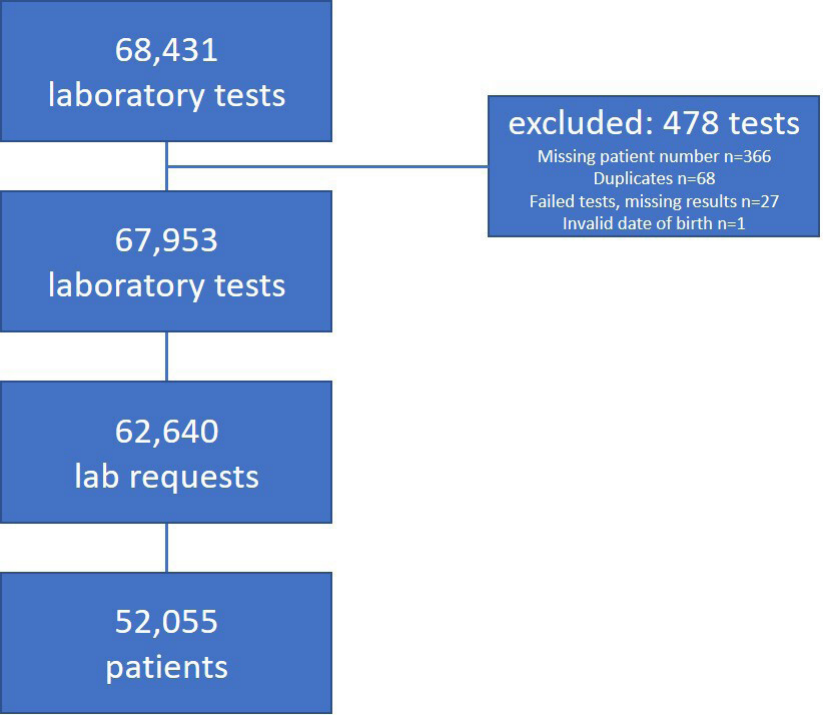
Exclusion of tests and number of remaining tests, requests and patients.

In total, 68 431 laboratory tests were extracted from the laboratory database; 478 tests were excluded from further analysis (Figure 1).

### Diagnoses

**Figure 2.**
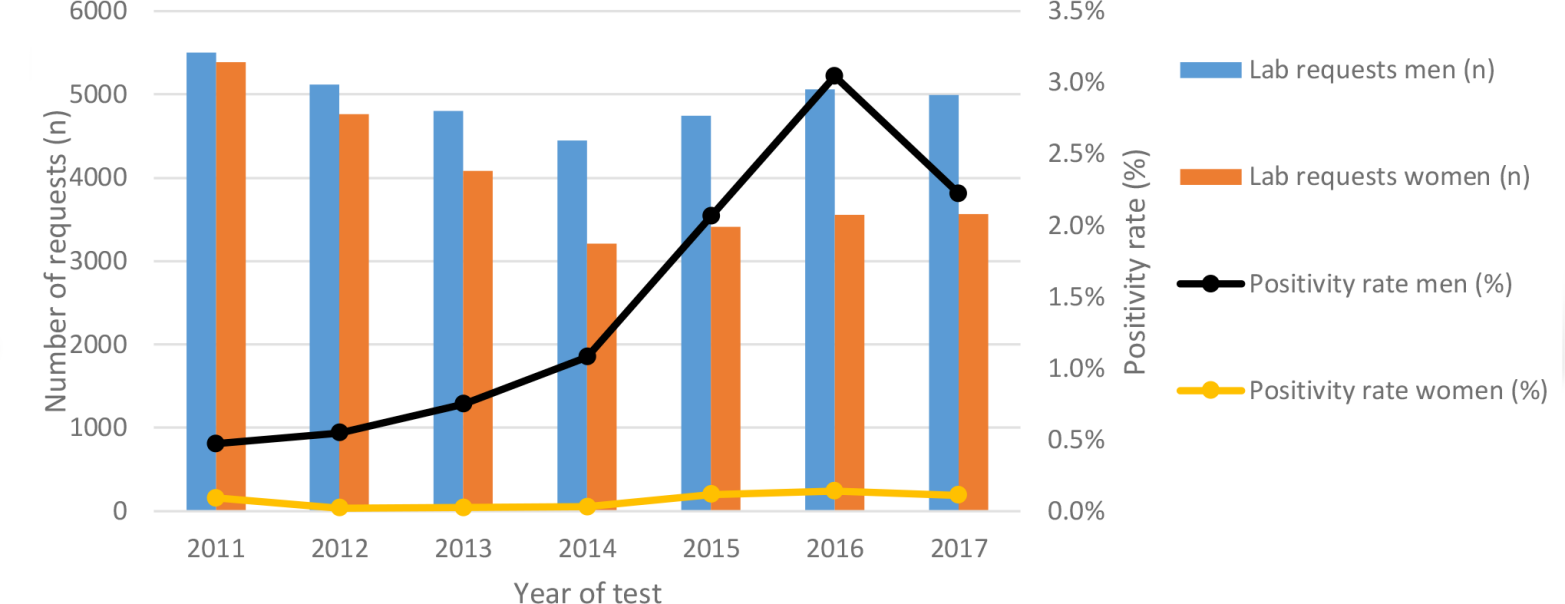
Annual number of lab requests and positivity rates in men and women. Positivity rate: number of syphilis diagnoses per number of lab requests. One lab request was defined as all laboratory tests in the same patient within a timeframe of 30 days.

Of 52 055 patients tested by GPs, 511 (1.0%) patients were diagnosed with syphilis (Table 1). Ten patients were re-infected, of whom one patient was re-infected twice, resulting in a total of 522 infections. Of all infections, 501 (96.0%) were in men; 21 infections were in women. The average positivity rate was 0.83%. Patients diagnosed with syphilis were significantly older than patients who tested negative (median age 44 years and 34 years respectively; *P*<0.001). The number of diagnoses in men increased from 48 in 2014 to 154 in 2016 and decreased to 111 diagnoses in 2017; the number of tests remained stable (Figure 2).

**Table 1.** Laboratory syphilis tests

Characteristic	****P**atients tested** ***n* =** **52 055**	**Syphilis diagnoses** ***n* = 522 **(positivity %**)**	****L**aboratory tests** ***n* =** **67 953**	**Positive laboratory tests** ***n* =** **6503**
**Sex**				
Male	28 090 (54.0)	501 (1.8)	39 060 (57.5)	5591 (14.3)
Female	23 963 (46.0)	21 (0.1)	28 891 (42.5)	912 (3.2)
Missing data	2	0	2	0
**Age** **years,** ^**a**^ **median (** **IQR** **)**	34 (27–44)	44 (35–52)		
<15	132 (0.3)	0	151 (0.2)	0
15–25	8478 (16.3)	33 (0.4)	9881 (14.5)	262 (*2.7*)
26–35	20 549 (39.5)	103 (0.5)	25 074 (36.9)	951 (3.8)
36–45	11 641 (22.4)	156 (1.3)	15 393 (22.7)	1649 (10.7)
46–55	7031 (13.5)	153 (2.2)	10 630 (15.6)	2050 (19.3)
>55	4213 (8.1)	77 (1.8)	6824 (10.0)	1591 (23.3)
**PCR**	N/A	N/A	617 (0.9)	106 (17.2)
**Anti-** ***T. Pallidum*** **(** **EIA** **)**	N/A	N/A	46 389 (68.3)	3095 (6.7)
Positive in the past^b^	N/A	N/A	N/A	1303 (42.1)
New positive^c^	N/A	N/A	N/A	85 (2.7)
**TPHA/TPPA**	N/A	N/A	17 125 (25.2)	1842 (10.8)
Positive in the past^b^	N/A	N/A	N/A	607 (33.0)
New positive^c^	N/A	N/A	N/A	30 (1.6)
Confirmation (immunoblot)	N/A	N/A	1042 (1.5)	926 (88.9)
VDRL/RPR (positive ≥1:4)	N/A	N/A	2780 (4.1)	534 (19.2)

^a^Age in years at first test for patients’ column, age at test for other columns. ^b^Some laboratories reported laboratory tests as 'positive in the past', even though it was the first lab request in this patient during the studied period. ^c^ New positive after an earlier negative test.

EIA = enzyme immunoassay. IQR = interquartile range. N/A = not applicable. PCR = polymerase chain reaction. RPR = rapid plasma reagin. TPHA/TPPA = Treponema pallidum haemagglutination assay/T. pallidum particle agglutination assay.VDRL = venereal disease research laboratory.

### GP’s contribution compared with annual reports of STI clinic in Amsterdam

**Figure 3.**
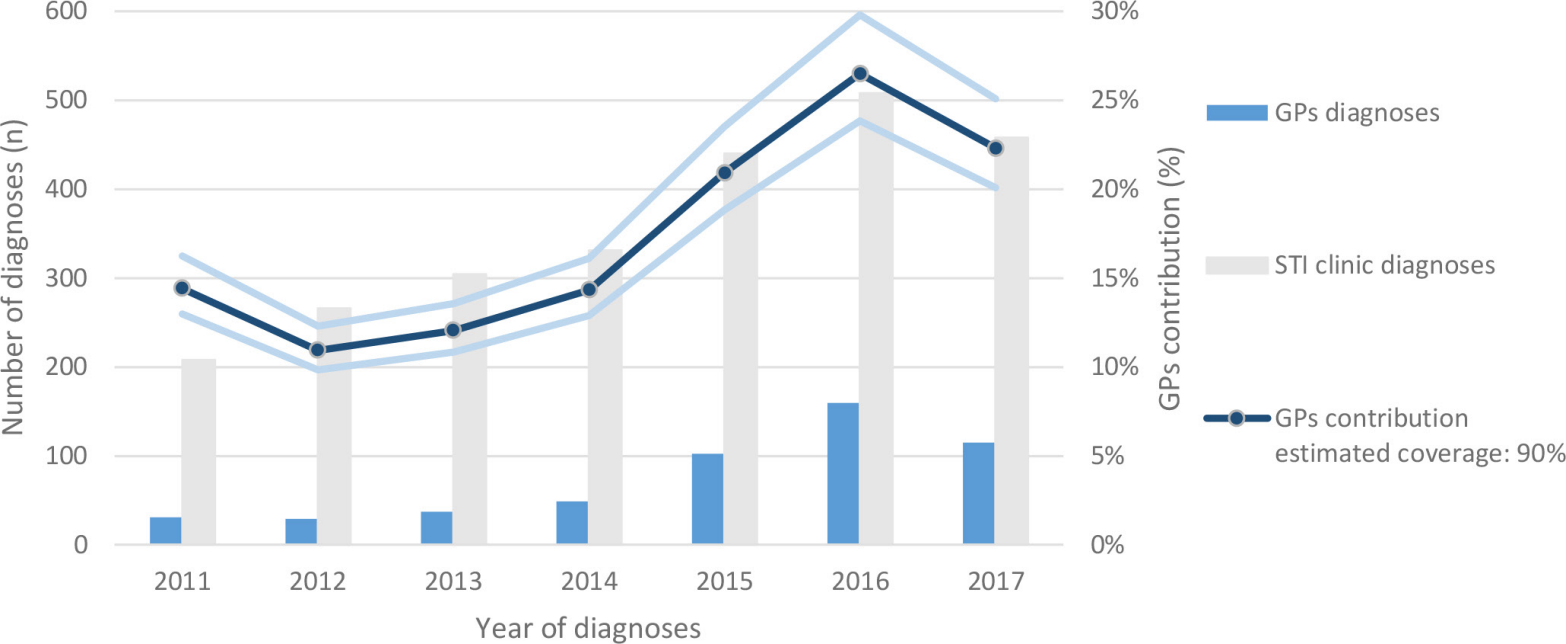
GPs and STI clinic syphilis diagnoses in Amsterdam. The number of syphilis diagnoses in Amsterdam between 2011 and 2017, shown for GPs and STI clinic. GPs’ contribution based on the extrapolated estimated 90% coverage of all GPs laboratory data in Amsterdam, with 80% and 100% coverage estimates as levels of uncertainty. STI = Sexually transmitted infection.

The number of cases of syphilis diagnosed at the STI clinic increased from 208 in 2011 to 508 in 2016 (Figure 3).^9^ In 2017, the number of diagnoses decreased to 458. Based on the authors' estimated 90% coverage, the GPs’ average contribution in diagnosing syphilis between 2011 and 2017 in Amsterdam is 19% (80%–100% coverage estimate: 21%–17%). The annual contribution of the GP increased from 11% in 2012 to 26% in 2016. When the authors assume the data represent 90% of an average of 811 500 inhabitants of Amsterdam, the annual incidence of syphilis in Amsterdam’s GP care is 10.2 per 100 000 inhabitants.

### Tests and positivity rates

Between 2011 and 2017, 62 640 lab requests were made (Figure 2), 7748 patients (14.9%) were tested at least twice. The number of lab requests slightly decreased until 2014, after which the number increased in men and remained stable in women. The positivity rate in men increased from 0.5% in 2011 to 3% in 2016. Patients aged 46–55 years tested positive most frequently; 147 men (3.1%) and six women (0.3%) were diagnosed with syphilis.

### Academic GPs’ network database

**Figure 4.**
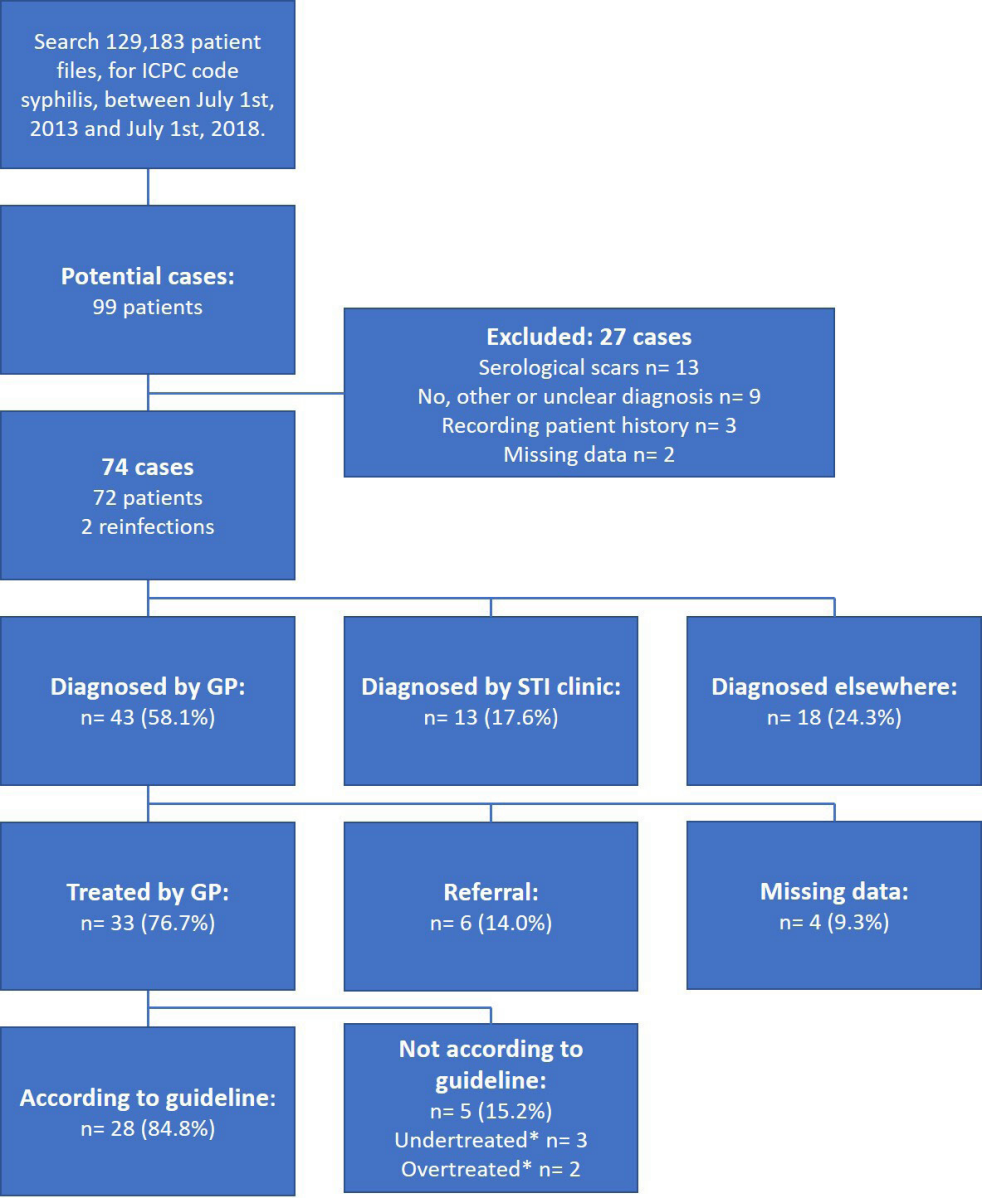
Potential syphilis cases and treatment of cases in general practice, separated by site of diagnosis. *Based on the stage of disease the first choice treatment of syphilis consists of one or three penicillin injections. Undertreated is when one penicillin injection was given when three were recommended by the guideline, and vice versa for overtreated. ICPC = International Classification of Primary Care. STI = sexually transmitted infection.

Seventy-four cases of syphilis were identified in the database; 43 of them were diagnosed by the GP (Figure 4). The annual incidence of syphilis diagnosed by the GP in this subset of Amsterdam’s GP network was 6.7 per 100 000 inhabitants. Thirty-three cases (76.7%) diagnosed by the GP were treated by the GP. Of these treated patients, 28 (84.8%) were treated with penicillin injections once or thrice, in accordance to the stage of disease. Three patients received one penicillin injection when three were advised (undertreated), two patients received three injections when one was advised (overtreated) by the GP’s guideline.^8^ Three patients (7.0%) were newly diagnosed with HIV and syphilis as concomitant STIs (Table 2).

**Table 2.** Characteristics of syphilis cases in general practice (*n* = 43)

Characteristic	**Frequency, n (%)**
**Sex**	
Male	36 (84)
Female	7 (16)
**Age, years**	
16–25	11 (26)
26–35	7 (16)
36–45	7 (16)
46–55	8 (19)
>55	10 (23)
(**Half) Year of diagnosis**	
1 July 2013–31 Dec 2013	5 (12)
2014	10 (23)
2015	9 (21)
2016	11 (26)
2017	8 (19)
1 Jan 2018–31 July 2018	0
**HIV status**	
Positive	8 (19)
Co-infection	3 (7)
Not recorded	32 (74)
**Stage of disease**	
Primary	10 (23)
Secondary	6 (14)
Early latent	3 (7)
Late latent	6 (14)
Neurosyphilis	0
Unknown^a^	17 (40)
Missing data	1 (2)
**Follow-up**	39 (91)
Missing data	4 (9)
**Referral**	6 (15)
Sexually transmitted infection clinic	3 (50)
Venereologist or internist	3 (50)
**Treated by GP**	33 (85)
1x penicillin	18 (55)
3x penicillin	15 (45)
**According to guideline** ^**b**^	28 (85)
Undertreated	3 (9)
Overtreated	2 (6)

^a^Unknown stage of disease or possible serological scar, which is clinically the same as late latent stage and requires 3x penicillin treatment.^b^ Based on the stage of disease the first choice treatment of syphilis consists of one or three penicillin injections. Undertreated is when one penicillin injection was given when three were recommended by the guideline, and vice versa for overtreated.

Eight patients first consulted the GP with symptoms of syphilis, but were referred for diagnostics to the STI clinic or a venereologist, and were thus diagnosed by the STI clinic or elsewhere, and were, according to protocol, excluded from analysis.

## Discussion

### Summary

The main objective of this study was to explore the role of the GP in diagnosing and treating syphilis in Amsterdam. It is assumed that syphilis is managed mainly by the STI clinic. The laboratory data showed that between 2011 and 2017 one-fifth of the syphilis cases were diagnosed by GPs. Furthermore, the contribution of GPs increased in recent years as well as the positivity rate. The study estimated the annual incidence of syphilis in Amsterdam’s GP to be between 6.7 per 100 000 and 10.2 per 100 000 inhabitants.

The majority (77%) of patients with syphilis diagnosed by the GP were also treated by the GP; the vast majority (85%) of them with the appropriate penicillin regimen.

### Strengths and limitations

One of the strengths of this study was the unique availability of a large laboratory database containing 90% of all STI diagnostics of all GPs in Amsterdam, providing accurate and extensive laboratory data. Therefore, it was possible to formulate a case definition based on laboratory tests. This is in contrast to other studies in Dutch general practice, which used data of the NIVEL, where case definition was based on ICPC-codes or surveys.^11,12^

Another strength is that overestimation was prevented, by manually checking every episode coded as syphilis in the academic GP network database, and excluding diagnoses made elsewhere. This way it was assured that a diagnosis was made by the GP themself and not by a different health provider.

A limitation was the absence of clinical information on symptoms and sexual behaviour, which might have led to misinterpretation and bias owing to the following issues. First, there is no single laboratory test that can accurately diagnose all stages of syphilis.^13^ Second, screening tests could be falsely positive because of cross-reactions with other treponema species. Third, VDRL/RPR titres can remain positive, even after successful treatment. The use of the strict case definitions might have led to both over- and underestimation. For instance, a positive VDRL/RPR test was defined as ≥1:4. In theory, someone who was infected and treated before the study period might have had a positive titre during the study period (and therefore be diagnosed) without having a new infection. On the other hand, patients with multiple positive tests, not meeting the diagnostic criteria, were not counted. In medical practice, test results are interpreted by the physician taking symptoms into account, as well as medical history and sexual behaviour, and could together meet the clinical diagnostic criteria for syphilis.

For the second part of the study, using the GPs’ database, syphilis case selection was based on searching for ICPC codes. The ICPC-system is prone to coding errors, since consultations have to be recoded based on laboratory outcomes. Consultations not being recoded, after receiving test results, could have led to missed cases. For instance, at first consultation an episode should be coded as ‘fear of STI’ or ‘skin rash’; after receiving test result this initial ICPC should be recoded to syphilis. Furthermore, when co-infections are present, every infection should be coded separately. For instance, when someone has a co-infection of gonorrhoea and syphilis, this syphilis infection could have been missed if only gonorrhoea was coded.

Another limitation is that the study only assessed whether the antibiotic treatment that patients received if treated in primary care was according to the existing guideline. Correct management of patients at risk for, or diagnosed with, syphilis needs a comprehensive approach, including testing, counselling, and partner notification.

The generalisability of the findings in this study is limited. First, Amsterdam is a high prevalence area for STIs. It is known that in multicultural and high urban density environments, STI prevalence is higher.^1^ Second, there is a large STI clinic in Amsterdam, leading to a proportionally limited role of the GP in STI care than in other parts of the Netherlands. Also, different districts of Amsterdam have different populations and STI prevalence. For instance, southeast Amsterdam, which consists of many non-western immigrants, is considered as a high prevalence area for STIs, such as chlamydia, gonorrhoea, and HIV.^1,10^ Syphilis is more common in the city centre where many MSM reside. The catchment area of academic GPs’ database network is located in these higher-risk areas.

Besides geography, data of the academic GPs’ network database might not be representative for the average GP. Since participating GP health centres choose to cooperate with the university, they might be more aware of and have more knowledge about current evidence and guidelines.

### Comparison with existing literature

In 2001, using a large national survey at primary care STI consultations, a prevalence of syphilis of 1.5 per 100 000 inhabitants was found.^12^ Between 2005 and 2007, based merely on ICPC coding of episodes in Dutch primary care, the incidence of syphilis was estimated to be 1.1 per 100 000 inhabitants, possible misclassifications included.^11^ In the study's population of Amsterdam’s GPs the incidence was between 6.7 and 10.2 per 100 000 inhabitants. The higher incidence in the study can be explained by the fact that GPs from a high prevalence area were studied and that overall incidences of syphilis nearly doubled. Similar incidences of syphilis in Belgian’s (Flanders) general practice were found to be 10 (6.8–13.9) per 100 000 inhabitants, between 2013 and 2014, where case definition was merely based on episode registrations.^14^

### Implications for clinical practice and research

The average Dutch GP takes care of 2095 patients; therefore, even in Amsterdam, syphilis is rare with only one case every 5 years per GP. Nevertheless the contribution of the GP in syphilis diagnoses in Amsterdam cannot be neglected and increased from 11% in 2012 to 26% in 2016. Also the positivity rate in men increased from 0.5% to 3%. A possible explanation for the increased contribution of the GP is the fact that in 2013 the guideline for GPs was updated and a decision tool for STI testing, including syphilis for risk groups, was developed.^8^ In 2014, the Amsterdam initiative for HIV elimination (https://hteam.nl/) started continuous education sessions for GPs related to STI and HIV testing, using the laboratory data as comparative feedback data during audit sessions for GP groups. These interventions might have achieved more attention for STIs in Amsterdam general practice and led to more targeted syphilis testing.

As syphilis is re-emerging, proper attention in primary care is warranted, especially since there are missed opportunities for testing for syphilis.^15^ The present study shows a substantial contribution of the GP in diagnosing syphilis, which brings along other responsibilities of STI care to the GP’s practice. Syphilis is known to be more common among MSM in specific high-risk groups, like MSM using drugs during sex (chemsex), and MSM using dating app or involved in group sex and/or visiting public sex environments (darkrooms, saunas, and so on).^2–5^ Knowing that these MSM groups are at high risk of HIV, patients with syphilis are offered PrEP against HIV at the STI clinic. For GPs, syphilis should be an indicator to offer PrEP, hepatitis B vaccination, frequent STI testing, counselling, and partner notification.

Quite unexpectedly, the present study showed that the majority of cases were treated by GPs themselves and were not referred. Although the study found that the overwhelming majority received adequate antibiotic treatment, the authors have no insight in correct and comprehensive management of these patients in primary care and further studies are warranted.

The re-emergence of syphilis has led to an increasing role of the GP in syphilis diagnostics. The idea that GPs barely contribute to diagnosing and treatment of syphilis is untrue for the studied urban population. However, for an individual GP, syphilis still remains a rare disease.
